# Influences on the physical and mental health of people with serious mental ill-health during the COVID-19 pandemic: a qualitative interview study

**DOI:** 10.1080/17482631.2022.2122135

**Published:** 2022-09-08

**Authors:** Elizabeth Newbronner, Lauren Walker, Ruth Wadman, Suzanne Crosland, Gordon Johnston, Paul Heron, Panagiotis Spanakis, Simon Gilbody, Emily Peckham

**Affiliations:** a Mental Health and Addiction Research Group, Department of Health Sciences, University of York, York, UK; b Independent Researcher, Clackmannan, UK

**Keywords:** Severe mental ill-health (SMI), mental health, physical health, COVID-19 pandemic, qualitative research, interview study

## Abstract

**Purpose:**

People with severe mental ill-health (SMI) experience profound health inequalities. The Optimizing Wellbeing in Self-isolation study (OWLS) explored the effects of the COVID-19 pandemic restrictions on people with SMI, including how and why their physical and mental health may have changed during the pandemic.

**Methods:**

The OLWS study comprised two surveys and two nested qualitative studies. Of 367 people recruited to the study, 235 expressed interest in taking part in a qualitative interview. In the first qualitative study eighteen interviews were conducted with a purposive sample of participants.

**Results:**

We identified six factors which influenced peoples’ health, positively and negatively: Staying Physically Active; Maintaining a Balanced and Healthy Diet; Work or Not Working; Daily Routine and Good Sleep; Staying Connected to Family, Friends and the Local Community; and Habits, Addictions and Coping with Anxiety Created by the Pandemic.

**Conclusions:**

Different aspects of lifestyle are highly interconnected. For people with SMI, loss of routine and good sleep, poor diet and lack of exercise can compound each other, leading to a decline in physical and mental health. If people are supported to understand what helps them stay well, they can establish their own frameworks to draw on during difficult times.

## 1. Introduction

There is growing evidence that the global COVID-19 pandemic and the restrictions put in place to contain it, has had a disproportionate impact on the physical and mental health of people with severe mental ill health (SMI; Burton et al., 2021; Gillard et al., 2021; Murphy et al., 2021; Peckham et al., 2022). Prior to the pandemic, people with SMI were already experiencing some of the most profound health inequalities of any group in society, with a mortality gap of between 15–20 years when compared to people without SMI (Hjorthøj et al., 2017). One of the biggest causes of this mortality gap is preventable physical diseases, such as cardiovascular disease and diabetes (Firth et al., 2019; Walker et al., 2015). Health risk behaviours can lead to an increase in physical disease. It is therefore concerning that recent research (Melamed et al., 2020; Peckham et al., 2021) has found that during the pandemic, people with SMI reported an increase in health risk behaviours such as smoking, harmful levels of alcohol consumption, low levels of physical activity and poor nutrition.

The pandemic restrictions and the wider impact of the disease on society, have also had major implications for mental health. People with existing mental health conditions are more at risk of experiencing social isolation, loneliness and loss of routine (Heron et al., 2022; Murphy et al., 2021; Wang et al., 2020) because of the pandemic restrictions. At the same time, access to the services which helped them maintain their mental health, often became more limited and many services were delivered partially or entirely remotely (Burton et al., 2021; Johnson et al., 2021; Newbronner et al., 2022). Gillard et al. (2021) used qualitative interviews (n = 49) to explore experiences of the pandemic for people with pre-existing mental health conditions (a quarter had SMI). Existing mental health difficulties were exacerbated for many people. People also experienced specific psychological impacts of the pandemic, struggles with social connectedness, and inadequate access to mental health services. New remote ways to access mental health care, including remotely-delivered and technology-enabled care, provided continuity of care for some but presented substantial barriers for others (Vera San JuaN et al., 2021; Sizer et al., 2022). Similarly, a qualitative study by BurtoBurton et al. (2021), involving 22 people with mental health conditions (the majority of whom had a diagnosis of depression), identified five pandemic-related factors contributed to deteriorating mental health: feeling safe but isolated at home; disruption to mental health services; cancelled plans and changed routines; uncertainty and lack of control; and rolling media coverage. The authors note that people with severe mental illness were particularly negatively affected.

Conversely, there is also evidence that during the pandemic some people with SMI took active steps to look after their physical and mental health. As in the population at large, they tried to stay physically active; took steps to maintain a daily routine; and found new ways to keep in touch with friends and their local communities. The study by BurtoBurton et al. (2021) highlighted five coping strategies which enabled people to maintain their mental health: previous experience of adversity; social comparison and accountability; engaging in hobbies and activities; staying connected with others; and perceived social support. The authors also suggest that some participants drew upon reserves of resilience and adapted their coping strategies in an effort to maintain their well-being. This finding is supported by Gillard et al. (2021) who note that some participants found new ways to cope and connect to the community. A scoping review by Murphy et al. (2021) drew similar conclusions. Several of the included studies reported that people with pre-existing mental health conditions were *“more likely to have past experiences of negative detriments of health (e.g., isolation, fear, unemployment), factors which are now similar to those associated with the pandemic”* (p388). The review authors go on to suggest that as a consequence, this group may have greater resilience and more established coping strategies, which helped them in the context of the pandemic. However, just as the negative impact of the pandemic has varied with factors such as age, ethnicity and social circumstances, so has the ability to adapt and cope (Fluharty & Fancourt, 2021).

The Optimizing Wellbeing in Self-isolation study (OWLS) was set up to explore the effects of the pandemic and the pandemic restrictions on people with SMI. The whole original study comprises of two surveys and two sets of embedded qualitative interviews. The study methods, procedures and questionnaires were developed by the research team in conjunction with our lived experience panel of six public contributors. This was an iterative process involving a series of meeting and discussions during the first three months of the pandemic. In this article we discuss the findings from the first set of qualitative interviews, which were conducted between September 2020 and January 2021. These were designed to explore key topics from the first survey, in greater depth. In this paper we focus specifically on participants perceptions of how and why their physical and mental health may have changed during the pandemic.

## 2. Materials and methods

At the start of the COVID-19 pandemic there was significant concern about the impact of the pandemic restriction on people with SMI. In order to rapidly examine this issue, a sample of people who had previously taken part in The Closing the Gap (CtG) study (a large (n = 9,914) transdiagnostic epidemiological clinical cohort) were invited to take part in the OWLS study. The composition of the CtG cohort has previously been described (Mishu et al., 2019). To be eligible to take part in the OWLS Study participants had to: be aged 18 and above; have documented diagnoses of schizophrenia or delusional/psychotic illness (ICD 10 F20.X & F22.X or DSM equivalent) or bipolar disorder (ICD F31.X or DSM-equivalent); and have capacity to consent to take part in the study. To reflect the diversity of the population, the sample of participant from the CtG Study selected to take part in OWLS was based on gender, age, ethnicity and whether they were recruited via primary or secondary care. They were contacted by telephone or letter and invited to take part in the OWLS study. The full methods of recruitment to the OWLS study have been previously described (Peckham et al., 2021).

At the end of the first survey (conducted between July 2020 and December 2020) participants were asked if they would be interested in taking part in a qualitative interview. Participants selected for interview (see Results section for details of the sampling process) were contacted by either phone or email and those who were still willing to be interviewed were sent a Participant Information Sheet and consent form. All the interviews were conducted by phone, with verbal consent being received and audio recorded prior to the interview. A topic guide for the interviews was developed and tested with members of our lived experience panel. The topic guide (see supplementary material) had four main sections, which mirrored the sections of the OWLS 1 Survey: changes in life since the pandemic restrictions; general health and wellbeing during the pandemic restrictions; access to health and other services; and staying socially and digitally connected. The interviews were conducted by EN (health services researcher) and lasted between approximately 30 and 90 minutes. They were audio recorded and transcribed (intelligent verbatim) in-house. Transcripts were checked for accuracy and anonymized by EN. Participants were offered a £15 shopping voucher to thank them for their time.

Transcripts were uploaded to NVivo 12 software for analysis. The concepts from the interview topic guide were used to provide an initial framework, within which the data was then analysed thematically (Braun & Clarke, 2014). One researcher (EN) conducted the initial analysis. A second researcher (LW) then randomly selected four transcripts and reviewed the coding of the data. Revisions to the coding structure and analysis were discussed with RW, and changes made by EN. The three main researchers brought their experience of health services research, health psychology, and mental health services delivery to the process of analysis. However, the full research team also met weekly to discuss emerging themes, develop findings and approve the final results.

Ethical approval was granted by the Health Research Authority North West—Liverpool Central Research Ethics Committee (REC reference 20/NW/0276).

## 3. Results

Between July and December 2020, 367 people were recruited to the OWLS study, and of these 235 expressed an interest in taking part in a qualitative interview. Eighteen interviews were conducted (between October 2020 and January 2021) with a purposive sample of participants. To achieve a sample that broadly reflected the age, gender, ethnicity and diagnosis of the OWLS participants as a whole, potential interviewees were grouped into a simple matrix. The groupings used in the matrix are the characteristics shown in Table 1. Participants were then drawn from 16 different areas of England (using the prefix of the participant identification code). Table 1 provides an overview of participant characteristics.

**Table 1.** Participant characteristics.

Characteristic	All Study Participants	Interview Participants
**Age in years**	n (%)	n (%)
18 to 45	150 (40.8)	10 (55.5)
46 or over	217 (59.2)	8 (44.5)
**Gender**		
Female	174 (47.4)	9 (50.0)
Male	187 (51.0)	8 (44.5)
Transgender	6 (1.6)	1 (5.5)
**Ethnicity**		
Minority Ethnic Groups	65 (17.7)	6 (33.3)
White British/Other White	302 (82.3)	12 (66.7)
**Diagnosis**		
Bipolar disorder	108 (29.4)	6 (33.3)
Schizophrenia or delusional/psychotic illness	188 (51.2)	7 (38.8)
Other SMI	23 (6.3)	3 (16.6)
Not recorded	48 (13.1)	2 (11.1)

At the time of the interviews, 11 participants lived alone and 8 were professionally active (i.e., in paid or voluntary work). Twelve were receiving support from mental health services, with the rest either being supported in primary care or by a private mental health practitioner or self-managing.

In presenting the results, we use quotations from participants to illustrate or bring to life the points being made. We have chosen to use pseudonyms rather than ID codes, in an effort to retain a sense of the people behind the data.

### 3.1 Changes in physical and mental health

In the first OWLS survey it was found that two thirds of participants felt their physical health had not declined (Peckham et al., 2021), and this was the case for many of the interview participants. A few even felt their physical health had improved slightly. Sometimes this was because they had been furloughed and had more time to look after their physical wellbeing, whereas others felt that keeping working, family support and an understanding GP had made the difference. A participant whose work meant that they were standing for much of the day but who was now furloughed reflected on their situation:

It’s been alright, you know, probably … I haven’t been on my feet all day, so I don’t know if that’s good or bad to be honest. I try and exercise occasionally. I don’t do as much as I should, but I have been, you know, trying to go for runs and stuff to make up for the fact that I’m not, you know, I’m used to being on my feet all day. So, yeah, I don’t think I’ve changed physically that much. I don’t think I’ve lost or gained any weight either which I know a lot of people have. Victoria

However, several participants described a general “slide” in their physical wellbeing, or a sense of “letting themselves go”, which was often associated with being less active, changing eating habits (e.g., “comfort eating”, eating more due to boredom) and weight gain, as the quotation below illustrates:

I mean in the bigger picture absolutely fine. I suppose like other people, in the circumstances, I have let myself go a bit. I’ve been so low that I’ve felt very dis-inclined to do any degree of exercise I normally do and I’m eating much more than I would normally eat and I have a vaping habit which has gone into the deep end really. I’m just vaping continually, I’m sort of comfort vaping. So I haven’t actually suffered any ill consequences in terms of illnesses or pain but I don’t feel very good about myself physically at the moment. Roisin

For a few participants, the deterioration in their physical health had been more dramatic. A number had physical health problems that had started before COVID-19. For some, the pandemic restrictions and reduced contact with health services, had led to a worsening in their condition and/or growing difficulties in managing it. A younger participant said that in the past two to three years their weight had increased from around 12 stone to nearer 30 stone. They were acutely aware that their health was declining but a combination of the difficulties created by their severe mental ill health, disrupted daily routine (including dietary patterns) and a poor response from health services, meant that it was not being addressed:

I think it’s because I don’t exercise whatsoever. I’m getting my food delivered. I never go out and socialise. So I’m not getting any daylight, any sunshine. I’m not socialising or getting any exercise. I’m eating round the clock, you know, eating through the night. Part of it is my medication that I take but part of it is comfort eating and depression … I have sleep apnea and nightmares, night terrors during the night. I’m waking up and eating sometimes and sleeping for days on end … Even though I told them [healthcare practitioners], you know, sometimes I was … I hate to say it but it was really embarrassing but wetting the bed and things like that. That has never happened to me in my life before until about a year ago and I told them my worries and I was feeling faint and dizzy. Simon

Another participant explained that they had bladder/kidney illness that meant he needed to urinate more frequently. They had a card, stating that they had a medical condition, which they could show when requesting to use a toilet, but during COVID-19 many places were still reluctant to let them use their facilities.

It’s really, really hard and obviously people are thinking of COVID-19 now and … they reject me to use the toilet, but I understand where they’re coming from. But my problem obviously is hard to explain and they’re never going to understand my problem. Even if I tell them the problem they’re still going to say, okay for health reasons we can’t give you access to toilet. So, I try to use street and road but obviously it’s embarrassing to pee on the street because people are watching you and sometimes, one or two times, people were swearing at me because of I’m peeing on the street and, you know, there was swearing, shouting and all this. Farooq

They went on to explain that they had been receiving regular counselling and support from a local community organization, but this had ceased during the pandemic. Without this support their mental health had begun to decline and was further affected by the practical and emotional difficulties created by their physical health problems.

Around half the interview participants described some worsening in their mental health. This mirrored the overall results from the OWLS 1 survey, in which 40% of participants reported that their mental health had declined (Peckham et al., 2021). Some participants described having “ups and downs” whilst others had experienced a slow and progressive deterioration. An older participant, who lived alone described how the pandemic restrictions and loss of face-to-face contact with their support worker had affected them:

I don’t want to eat. I don’t want to get dressed because I don’t see anybody. I feel why should I get dressed? I’ve been in a nightdress and housecoat since the lockdown … It’s affected me really badly, you know. I know it’s affecting everybody isn’t it but I mean I used to like going out and doing my own shopping but now I don’t want to now … I’ve had to be put on anti-depressants. Alison

Another participant explained that the isolation of the first lockdown that had led to quite a rapid deterioration in their mental health but fortunately this had been picked up very quickly by their GP:

So from the first lockdown after about two months I was starting to get hallucinations. Things were going missing in my house and I thought that someone was breaking in to take them as trophies. I was seeing billboard posters and thinking there were targeted at me, someone was trying to send a message to me, specifically from an ex-boyfriend who wanted revenge. So I phoned my doctor about something else, completely different and she said, how are you otherwise? And I said, well I lost something today and I thought someone, you know, broke in to steal it and she said, ‘ah that sounds a bit like your weird thoughts that you had 4 years ago, I think we should talk to a psychiatrist’. Aaliya

The interaction between peoples physical and mental health was a common thread throughout many of the interviews. A younger participant reported that early in the pandemic they had been ill with what may have been COVID-19, and after that they began to struggle with her mental health:

I was managing a bit more up until May and then I was ill and that really affected my lung capacity and then I didn’t have the energy to exercise. That’s possibly one of the reasons my mental health started going downhill. Sarah

The extract below vividly illustrates how during the pandemic, people with co-existing mental and physical health problems found it particularly difficult to maintain their health:

I tried but it’s not easy to, you know, manage those things because once you got mental health issues, sometime you don’t think straight and sometime you don’t think about your health, you know. I don’t know, individuals have a different way they deal with it but myself it’s not easy to stay healthy, eat healthy or sleep healthy. As I said I’ve got insomnia. Insomnia is really one of the illnesses. I’m more suffering because if anything happens in the day it stays in my mind all night and keeps me, you know, talking to myself and then end up taking sleeping tablets because I’m on sleeping tablet as well and anti-depression and insomnia, anxiety medication. Farooq

### 3.2 Factors influencing peoples’ health

In the following sections we explore in more depth the factors influencing peoples physical and mental health. Our findings are grouped into six themes. Within each we discuss the impact of the pandemic restrictions, both positive and negative. We also consider the steps people took to try and look after their health and interconnections between the themes.

### 3.2.1 Staying physically active

It was clear that, in line with the general population, the pandemic restrictions had made it more difficult for some participants to stay physically active. Those who normally took part in regular, organized activities such as team sports, exercise classes, swimming and gym sessions, were particularly affected. Below, two participants describe how the loss of these activities had affected them:

Normally when this isn’t happening, I’m a member of a sports club and I exercise two, three, four times a week. Exercise is really helpful but I haven’t been able to do that during the pandemic … plus the social side of it, that really helped as well. Sarah

I mean one thing that I have lost at the moment is my gym because I used to find going to the gym after work and that, do a couple of hours workout really does help but because the gyms are shut, I’ve put weight back on as well! Philip

However, several participants had found alternatives. One had replaced swimming with running and noted that it helped: *“even out my breathing and makes me forget about paying too much attention to my body”* (Victoria). Others were going out for regular walks or cycle rides: “*I’m always walking. I bought me self a pair of trainers. I’m always out. I’ve got to get out. I cannot sit”* (Karen). One was doing exercise classes via Zoom whilst another explained that they and their partner had taken in a foster dog and this had helped them stay active. Both the above quotations reinforce the point that for many people physical activity was about maintaining their mental as well as physical wellbeing.

### 3.2.2 Maintaining a balanced and healthy diet

Several participants, especially those with co-morbid physical health problems such as inflammatory bowel disease and colitis, talked about making a conscious effort to eat a healthy diet. One explained that just before COVID-19, they had started trying to eat a healthier diet: *“I was eating sort of takeaways and it was giving me bad indigestion. But I have become real health conscious”* (Trevor). Importantly, they went on to say that they had managed to maintain this change: *“No takeaways for over a year and a half now and I’ve started having whole, particularly whole foods, no junk food”*. In fact, the pandemic restrictions had helped this participant, as they felt less anxious going to the supermarket when there were fewer people there.

However, many were finding it difficult to maintain a healthy diet. Sometimes this was caused by practical problems such as getting food deliveries when they were self-isolating or struggling to go shopping on their own. For others, a shift to convenience and energy-dense food it was linked to motivation:

I’m not eating very well either. I tend to eat a lot of junk food at the moment, so [I’m] trying to get that sorted out now … I think it’s motivation. Just feeling a bit down and rubbish generally. I tend to want comfort food and I don’t have the energy to cook as much. Sarah

Overeating and associated weight gain was also an issue of concern. Sometimes this issue had been long standing and the pandemic restrictions had made the shift to energy dense convenience food more pronounced. However, for others the loss of routine and being at home more were important factors.

### 3.2.3 Work or not working

In the first OWLS survey, just over a quarter of respondents were “professionally active”, with 4.1% being furloughed or on paid leave. A further 10% reported a change in either working hours and/or their duties (Peckham et al., 2022). A much higher proportion (44.4%) of the interview participants were professionally active and so their experiences may be slightly different to the whole survey group. Nevertheless, the two quotations below illustrate the impact these work changes had on peoples’ physical and mental health:

Sort of normally I would be really busy at work, on the go all the time … always walking. So to go from that 6 days a week to then maybe only do that 2 or 3 and then working at home the rest, so a massive change in my activity. Jenny

I didn’t like the working from home, where it was solidly working from home that was what brought on my mental health, triggered my mental health problem again. Yes, and my employer was very supportive and said I could work a few days a week after the first lockdown … they thought that contact with people, my colleagues, who are also coming in, not all of them but about half of them are coming in, they thought that might help and it’s helped tremendously. Aaliya

Those who had been able to go to work (including voluntary work) during the pandemic restrictions generally felt it had helped them maintain their wellbeing. For some it was about keeping busy and active: “*Another good thing is keeping busy, like routine busyness sort of thing … I do a lot of walking. I do probably on average 15,000 to 25,000 steps a day. I mean that’s at work … because I walk around the building*” (Philip). For others it was the social contact that they valued. We discuss this further below.

### 3.2.4 Daily routine and good sleep

A number of participants, especially those in the younger age group, were very aware of the importance of having a routine, including good sleep patterns, for managing their mental health.

I think one of the most of important things to help my condition is maintaining a proper sleep schedule … I suppose mindfulness has been quite good in terms of sleep, like making sure you get that good sleep no matter what, so like listening to meditation tracks and all that. Ryan

They talked about making a conscious effort to maintain these things during the pandemic restrictions. For some people, paid or voluntary work had provided a core structure for them to build their routine around: “*It’s probably the routine, you know, there was the routine I was getting up, getting dressed, getting ready to go to work and everything”* (Karen). Being out of work, furloughed or working from home often disrupted this but many participants took active steps to preserve a routine. One participant who had recently been diagnosed with ME, had temporarily given up work but nevertheless tried to keep to their sleep routine:

I have a set routine where at 9 o’clock my phone goes onto silent, it sounds really daft like little messages coming through say on Facebook or other social media but I don’t answer the phone … I’ll go to bed sometimes between 10 and 11 but the television always gets turned off at 11 o’clock and I’d say most nights I’m asleep between half past eleven and midnight and then, you know, I’m awake between half seven and quarter to eight. So that’s my routine and it’s just the little things but they do help me. Bella

However, others were finding it very hard to sustain a good sleep pattern and this was having a detrimental impact on their wellbeing:

My sleep pattern is really bad. I’m not sleeping regularly and then I tend to fall asleep on the sofa which is not healthy and then go to sleep really late, go to bed really late. I’m struggling with regular sleep and I know that’s not helpful. Sarah

This participant felt that their poor sleep pattern was linked to a deterioration in their diet and the fact that they were no longer able to take exercise with their sports club. Their experience highlights how interconnected different aspects of lifestyle are, and how for people with SMI in particular, loss of routine and good sleep, poor diet and lack of exercise can compound each other.

The change in routine brought about by the pandemic restrictions also gave some people more time and space to think about their situation. This had both negative and positive consequences. One participant whose mental health had deteriorated, explained: “*I had more opportunities to face my problems. Like before there was more distraction and a lot more to do and then now there were no distractions and I literally had to face my problems”* (Saadiqa). However, another said that having space to reflect and re-evaluate things had been helpful:

I think actually for me, as somebody that always feels that I need to be on the go and doing things, and I’m not doing enough and I’m not good enough and I’m not pushing enough, it’s actually taught me over this period to be able to sit down and read my book for an hour or watch TV because I can’t physically do anything else. So in some respects that’s been really good because I’ve been able to re-evaluate what I actually want to spend my time doing. What I need to spend my time doing for my own mental health and kind of prioritising things a bit differently. Jenny

### 3.2.5 Staying connected to family, friends and the local community

Nearly two thirds of the interview participants lived alone. A few had little or no support from family and friends and relied on mental health and voluntary sector services for their social contact. During the most severe pandemic restrictions, this support became more limited and was often only available by phone, which led to some participants feeling extremely isolated and lonely. However, others had been able to keep in touch with family and friends, sometimes embracing new ways to do this like Zoom and WhatsApp video calls or computer games played with groups of friends: *“I’d say I’ve played more computer games, I would say that’s like a social thing as well, it’s kind of like chatting to people.”* (Ryan). Conversely, people sometimes found not having to socialize was a relief:

*I’ve quite enjoyed the lockdown because if I’ve had times when I’ve made arrangements with people and then I don’t really want to follow through with them because I just feel so awful … because of the lockdown and being in Tier 3* [the highest level of restrictions in the UK] *I’ve not had to make those commitments*. Bella

As was noted earlier, paid and voluntary work played an important part in enabling people to stay connect socially and to the community. A participant, who worked in a charity shop described how the reopening of the shop had made them feel: *“It was like a lot of the old customers came back and they were so, ‘oh I’m so glad you open’. I spent more time talking to them than selling things!”* (Karen). Another explained that even interacting with people at a distance or on the phone was beneficial, added to which volunteering gave them a sense of being useful:

In the first lockdown, you know, when it first all happened, I was quite involved in a mutual aid group down here and that was quite helpful because I think one of the things you do lose without being able to go into town and go and see people is that sense of like community, you know, especially because I live in quite a small town so you do tend to know everyone. Being involved in that I did find really helpful, because you were both seeing people, even at a distance, being very careful dropping food to them whatever but also just constantly on the phone to people, you know. Victoria

### 3.2.6 Habits, addictions and coping with anxiety created by the pandemic

It was clear that the pandemic restrictions had exacerbated some participants health risk behaviours or led to old addictions resurfacing. Several said they were smoking or vaping more or had started smoking again. Sometimes this was related to boredom and the loss of other activities and distractions:

I mean I did start smoking again … and that’s been quite a disappointment because I gave up for about three years. That’s not brilliant … I think it was just missing the things to do that I usually did. I wasn’t going down the pub. I wasn’t meeting up with people so and I just became a little bit frustrated with that and started smoking again. Andrew

However, for some participants it was a means of coping with anxiety:

I did start again probably last month. My anxiety got worse and I do tend to do that. I’ll stop and then, you know, if my mood will change, I’ll start again. I have started straight back onto vaping, not cigarettes because I can literally try and minimise the damage. But yeah my nicotine habit is quite up and down. Victoria

One participant explained that they had previously had a drug addiction. Although they had been “clean” for several years, the COVID-19 pandemic triggered their addition once again. The extract below from their interview describes their experience but also illustrates how they found a way to tackle the problem themselves with the support of peers:

At the start of the very, very first lockdown I did relapse slightly with pain-killer addiction … before I was diagnosed with bipolar disorder, I was an active drug addict and then I got clean … the start of the lockdown it really threw me. Like when, you know, with all the news broadcasts, it seemed like it was this invisible weapon like a war and it sounded like the country was going to war and I was sat watching it and I started to freak out and I did relapse … .I started attending meetings again because I used to go to Narcotics Anonymous meetings a long time ago which is what helped me get clean in the first place … the town that I live in has a really good community of recovering addicts and I was just welcomed back into the group basically and I wasn’t the only person who’d been doing really well and had been clean and been sober and who had relapsed because of this and it was just momentary. Bella

Interestingly, very few participants reported that their alcohol consumption had increased. One person said that Christmas 2020, had been very a very difficult time for them and they had a short period of binge drinking. However, like the participant above, they had sought support from members of an Alcoholics Anonymous group they had belonged to in the past and had been able to bring her drinking under control. Two others said that they had started drinking alcohol again but not to excess. A small number of participants also talked about their high consumption of energy drinks. One said described themselves as *“not a good eater”*, and so they were drinking Lucozade instead of eating regular meals. Another explained that they had been drinking four to five litres of energy drink each day. They had cut down to one to two litres but had been unable to stop drinking them completely: *“I’ve cut right down but I couldn’t stop straight because my body would go into shock with the, is it sugar intake or something like that”* (Philip). This is concerning, as high consumption of sugary drinks is regarded as an important indicator or proxy for very poor diet.

The interviews revealed that people had found a variety of positive ways to manage the anxiety created by the pandemic. As in the general population, some people had turned to hobbies and interests, such as art and music:

I mean I quite like, you know, like Channel 4 did that art club thing, like I think projects like that where it gets people engaged in doing things that they might not otherwise do but can be therapeutic but in an accessible format like on TV is good. (Ryan)

Several people described limiting how much news they watched or read and making a conscious decision to watch TV programmes or read things that were positive or uplifting.

## 4. Discussion

It is now clear that the restriction introduced to control the COVID-19 pandemic, exacerbated the mental health problems experienced by people with pre-existing mental health conditions (Byrne et al., 2021; Gillard et al., 2021; Murphy et al., 2021). However, an increase in health risk behaviours (Peckham et al., 2021) and worsening in physical health (Melamed et al., 2020) is of equal concern, especially for people with severe mental ill health. A number of qualitative studies have begun to shed light on the possible causes of this deterioration (Burton et al., 2021; Gillard et al., 2021; Lyons et al., 2021). The findings from this study add to our understanding of this issue but also highlight the ways in which people with severe mental ill health have looked after their health, by drawing on their own resources, strategies and support networks.

The bidirectional and synergistic relationship between physical and mental health was a thread running through our findings. Those with existing physical health problems often found it more difficult to manage them during the pandemic, in part due to changes in services and support or loss of routine, and this in turn had a detrimental effect on their mental health. We suggest that there is a small but significant group of people with mental and physical multimorbidity who are particularly vulnerable. For them, disruption to their lives and/or loss of services, whatever the cause, can be a tipping point that leads to significant decline in their health and wellbeing. This is a lesson that goes beyond the COVID-19 pandemic and reinforces the importance of services identifying and supporting this vulnerable group.

Just as in the general population, many of the things that people normally did to maintain their health, such as staying physically active or maintaining a healthy diet, were more difficult during the pandemic. However, as a number of other studies have found (Gillard et al 2021; Burton et al., 2021; Gonzalez-Blanco et al., 2020), people who lived alone or were more reliant on support from services for shopping and other routine activities, experienced greater difficulties. They often struggled with motivation but lacked the support from family and friends that provided external motivation and bolstered other participants in the study. The loss of routine caused by changing work arrangements such as being furloughed or working from home, or the suspension of volunteering activities, was also an important change for many. As other studies have found, it had implications for both mental and physical health (Burton et al., 2021; Murphy et al., 2021). However, younger participants in particular, were often aware of the importance of routine, including good sleep patterns and took active steps to maintain these things. Similarly, those people who experienced a recurrence of addiction issues during the pandemic, revisited strategies and sources of support that had helped them in the past. This suggests that if people are supported to understand what helps them stay well, they can establish their own frameworks to draw on during difficult times. However, neither the life experiences of people with SMI, nor the things that help them to stay well are homogeneous, and so the support provided and the approaches used need to be individualized.

Our findings highlight just how interconnected different aspects of lifestyle are, and how for people with SMI in particular, loss of routine and good sleep, poor diet and lack of exercise can compound each other, leading to a decline in both physical and mental health. As the COVID-19 pandemic recedes, we must not lose sight of this.

## 5. Conclusions

As in the general population people with SMI had differing experiences of the pandemic restrictions with some people being more severely affected than others. Maintaining a daily routine and a healthy diet along with good sleep and talking part in physical activity were all reported to be protective factors in terms of both physical and mental health. Supporting people with SMI to achieve these goals may help to protect against worsening health in future crises, whether national or personal.Further research is needed to understand how to best support people with mental and physical multimorbidity, in particular the place of practical and motivational support for those who live alone or have poor support networks.

## 6. Limitations

Only 18 interviews were conducted however they constituted a good cross section in terms of age, gender and ethnicity. A higher proportion (almost half) of interview group were professionally active compared to the cohort as a whole (just over a quarter). This could be an indication that the participants who took part in this study were generally less unwell than the overall study population. However, several participants had experienced significant physical and mental health problems, suggesting that the views of those who were less well were still represented. In addition, pandemic restrictions in the UK varied over the study period but some restrictions were in place throughout the duration of the study.

## Supplementary Material

Supplemental Material
