# Supplementary material for: Influences on the physical and mental health of people with serious mental ill-health during the COVID-19 pandemic: a qualitative interview study
Source: Int J Qual Stud Health Well-being. 2022 Sep 8;17(1):2122135. doi: 10.1080/17482631.2022.2122135 (PMC9467576; doi:10.1080/17482631.2022.2122135)
Supplement: Supplemental Material [file ZQHW_A_2122135_SM0417.docx]

**Interview Schedule for OWLS Qualitative Study**

**Introductory Notes:**

- Thank the participant for helping us with the OWLS study
- If necessary, briefly remind them of what the study is about and note that we are interested in how things have been for them since the pandemic restrictions started in March 2020
- Explain that the interview is likely to take 30 to 40 minutes and check that it is still a good time to talk to them
- Explain that we need to confirm their consent to take part in the interview
- Turn on the recorder
- Go through consent form
- Turn off the recorder
- Check if they have any questions or concerns before starting the interview

*** TURN ON THE RECORDER ***

**A: Changes in life since the pandemic restriction**

1. In what ways, if at all, is your life different since the pandemic restrictions started back in March?

a) Have there been any changes in your living arrangements? Would you mind telling me about that?

b) Has your work situation changed or stayed the same? If yes, how have you found that?

**B: General health and wellbeing during the pandemic restrictions**

2. Can you tell me how your health has been during the pandemic restrictions?

Prompts:

- Physical health and/or general wellbeing? Same/Better/Worse?
- Mental health? Same/Better/Worse?
- Explore reasons for responses (positive and negative) if offered and/or pick up in Q3 and Q4

3. Are there any things you normally do to try and look after your health (e.g. exercise, healthy eating, getting enough sleep)?

a) Have you been able to keep up these habits during the pandemic restrictions?

If necessary prompt for: type of habits (e.g. keeping a routine, finding different ways to keep active); what has helped (e.g. support of friends/family, pets)

4. Most of us have some habits that might not be so good for our health (e.g. smoking, not keeping active). Would you happy to tell about any you might have and if/how they’ve changed during the pandemic restrictions?

If necessary prompt for: type of habits (e.g. smoking a bit more, being less active, or eating more unhealthy foods); reasons for change

**C. Access to health and other services**

5. Have you used health services for a physical health problem since the pandemic restrictions started?

If no:

6. Was that because you didn’t need them or for another reason (e.g. didn’t want to use them during the pandemic; couldn’t get access)

If yes:

7. Do you mind me asking, was that for a long standing health problem or something new?

8. Could you tell me about your experience of using physical health services during the pandemic restrictions?

1. What did that involve?
2. In what ways was that different to what usually happens (e.g. phone instead of face to face; more limited range of services)?
3. Were you given information about how services might be different?
4. How did you find the changes?

9. Have you used health services for mental health problems since the pandemic restrictions started?

If no:

10. Was that because you didn’t need them or for another reason (e.g. didn’t want to use them during the pandemic; couldn’t get access)

If yes:

11) Could you tell me about your experience of using mental health services during the pandemic restrictions?

1. What did that involve?
2. In what ways was that different to what usually happens (e.g. phone instead of face to face; more limited range of services)?
3. Were you given information about how services might be different?
4. How did you find that?

12. Have there been any changes to services that you would like to see continue once the pandemic restrictions have been lifted?

**D. Staying socially and digitally connected**

13. Could you tell me a bit about any support you’ve had from family and friends since the pandemic restrictions started in March?

If possible, explore whether this is more/same/less than before the pandemic and if/how this has made a difference

14. More generally, has your social life changed as a result of the pandemic restrictions. Is better, worse, the same, different (e.g. more difficult)?

a) Could you tell me in what ways it has changed?

b) Could you tell me a bit about why it has changed (e.g. not wanting to mix with people too much; easy to keep in contact via the internet etc.)?c) Have these changes affected your health and wellbeing in any way?

15. Before the pandemic restriction, did you talk to your family/friends via the internet (e.g. on Skype, WhatsApp)? Has this changed during the pandemic?

16. In what other ways, if at all, do you use the internet in your everyday life (e.g. online shopping, making bookings/appointments, social activities, following news and events?

**E. Closing thoughts**

17. When you think about how things might go over the next few months, how does this make you feel?

18. Is there anything that you think I should have asked you about, but haven’t and/or is there anything else you would like to tell me or ask me?

**Closing Notes:**

- Thanks the participant for taking part in the interview
- Check if they have any final questions or concerns
- Add a friendly question/comment e.g. what have they have planned for the rest of the day or enjoying/avoiding the weather etc.
